# Changes in Orbital Volume following Reconstruction with Alloplastic Materials in Patients with Orbital Trauma 

**DOI:** 10.30476/dentjods.2025.104766.2549

**Published:** 2026-03-01

**Authors:** Mohammadsaleh Khaghaninejad, Mohammad Dehghani, Farhad Ghorbani, Banafsheh Salehi

**Affiliations:** 1 Dept. of Oral and Maxillofacial Surgery, School of Dentistry, Shiraz University of Medical Sciences, Shiraz, Iran.; 2 Undergraduate Student, School of Dentistry, Shiraz University of Medical Sciences, Shiraz, Iran.

**Keywords:** Orbits, Orbital Fracture, Plastic Surgery Procedure, CT Scan, Reconstructive Surgery

## Abstract

**Background:**

Internal orbital reconstruction is a commonly performed procedure in management of zygomaticomaxillary complex (ZMC) fractures; however, it is not indicated in all cases. In management of orbital trauma, surgeons should not only correct the apparent defects, but also must reinstate the function of the orbit.

**Purpose:**

This study aimed to assess the changes in the orbital volume following reconstruction with alloplastic materials in patients with orbital trauma.

**Materials and Method:**

This retrospective study evaluated all patients with unilateral orbital fracture presenting to the Oral and Maxillofacial Surgery Department of Rajaie Hospital (from 2013 to 2016, Shiraz, Iran) who underwent corrective surgery. The computed tomography (CT) scans of patients were analyzed by Volumetry software to quantify the change in the orbital volume after surgical reconstruction of the orbital floor with alloplastic materials compared with the sound contralateral orbit. Data were analyzed using the Chi-square test and Student t-test (alpha=0.05).

**Results:**

Significant differences were found in the volume of the traumatized orbit with sound contralateral orbit and the orbital volume after corrective surgery (p Value< 0.05). Corrective surgery was successful in all patients, and the orbital volume after treatment had no significant difference with the volume of the sound contralateral orbit (p> 0.05).

**Conclusion:**

Corrective surgery with alloplastic materials can successfully regain the orbital volume in patients with orbital floor fracture, with no significant difference with the volume of the sound contralateral orbit.

## Introduction

Orbital trauma is commonly found in trauma patients. It is often associated with functional complications such as diplopia, blurred vision, blindness, and inability to move the globe, as well as appearance-related complications such as enophthalmos, dystopia, and asymmetry. Several materials such as allografts, autografts, and alloplastic materials may be used for reconstruction of traumatized orbital bony walls [ 1 - 2 ]. Because the orbit’s precise three-dimensional contours are crucial for globe position and soft-tissue support, reconstruction remains technically demanding, and there is still no consensus on optimal timing, surgical approach, or implant choice [ 3 - 5 ].

The zygomaticomaxillary complex (ZMC) fractures often include the orbital floor fracture. The size and extent of the orbital floor fracture may vary from a crack line to shattering of the entire orbital floor and fracture of the medial and lateral walls. The majority of the ZMC fractures do not cause herniation of the orbital content into the sinus or enophthalmos, and such complications occur in only a small percentage of orbital trauma patients. It has been reported that 47% of patients with zygomatic fractures and two-thirds of those with ZMC fractures have symptoms of orbital floor complications [ 2 , 6 ]. 

Some, but not all, surgeons believe that examination of the internal orbit should be routinely performed intraoperatively in surgical correction of ZMC fractures. Computed tomography (CT) can be of great aid for this purpose [ 6 - 7 ]. Some operating rooms are equipped with a CT machine for intraoperative use. Thus, when the preoperative CT scan cannot reveal the defects, intraoperative CT may be indicated after reduction of the ZMC for assessment of the internal orbit. Accordingly, the surgeon may decide about the need for reconstruction of orbital walls [ 8 - 9 ]. 

Previous studies have investigated the relationship between orbital volume changes and postoperative globe position, particularly enophthalmos, with volumetric assessment emerging as a key predictor of outcomes [ 10 ]. A systematic review and meta‑analysis by Murray‑Douglass *et al*. [ 11 ] reported a pooled correlation coefficient of r= 0.71 between increased bony orbital volume and post‑traumatic enophthalmos, indicating that volumetric expansion accounts for approximately 50 % of the variance in globe displacement. Prospective longitudinal studies have demonstrated that postoperative orbital volume correction correlates with globe malposition, with Snäll *et al*. [ 12 ] reporting a 40% incidence of globe malposition at six months despite satisfactory volumetric restoration, highlighting the influence of fracture size and soft-tissue prolapse. However, some retrospective analyses have questioned the long-term predictive value of volume change alone for late enophthalmos and diplopia, suggesting that fracture location, soft-tissue atrophy, and orbital fat scarring also play significant roles in postoperative outcomes [ 3 ].

Internal orbital reconstruction is a commonly performed procedure in management of ZMC fractures; however, it is not indicated in all cases [ 13 - 14 ]. In management of orbital trauma, surgeons should not only correct the apparent defects, but also must reinstate the function of the orbit. Considering all the above, this study aimed to assess the changes in orbital volume following reconstruction with alloplastic materials in patients with orbital trauma. 

## Materials and Method

This retrospective study evaluated all patients with unilateral orbital fracture presenting to the Oral and Maxillofacial Surgery Department of Rajaie Hospital (from 2013 to 2016, Shiraz, Iran) who underwent corrective surgery. The sample size was calculated according to the results of previous studies [ 4 ] and using G-Power version 3.1 software at the alpha level of 0.05; the power of the test was 80% and the effect size was 0.4. A total of 73 patients who met the inclusion criteria were enrolled in the study. The study protocol was approved by the Ethics Committee of the Shiraz University of Medical Sciences with the code of IR.SUMS.REC. 1395.S1154, and all patients signed informed consent forms prior to their surgical procedure and also prior to study enrollment. 

The inclusion criteria were unilateral orbital fracture requiring corrective surgery and availability of preoperative and postoperative CT scans of patients (retrieved from the hospital archives). The exclusion criteria were orbital reconstruction with materials other than alloplastic materials, fracture of more than one orbital wall, patients with craniofacial deformities causing asymmetry, and patients with blindness following trauma and subsequent orbital enucleation. In our center, patients typically undergo surgical intervention within 14 days of the traumatic event. The timing of surgery depends on clinical factors such as the severity of periorbital edema and the need for other medical interventions by related departments.

Before surgery, all patients underwent 3D CT scans using the Emotion Somatom CT scanner (Siemens) while positioned in a standardized supine posture. After clinical diagnosis, all patients were examined by the same ophthalmologist for corrected visual acuity, diplopia, enophthalmos, and hypoglobus, along with a detailed medical history. The same CT scanner was used for post-operative imaging, and the study was based on the comparison of these pre- and post-operative standardized CT images.

The transcaruncular approach and inferior subciliary incision with lateral canthotomy were performed in all patients under general anesthesia. Through the incisions, minimal subperiosteal dissection was carried out up to the bony rim of the orbital floor and medial wall fracture. Intraorbital soft tissues that had herniated into the paranasal sinuses were returned to the orbital cavity. The length and width of the defect of the orbital floor fracture were measured intraoperatively, and the first titanium mesh plate was shaved and shaped referred to the contralateral orbit. The amount of reconstruction required was assessed based on the preoperative CT scan and direct intraoperative evaluation of the defect. The medial side of the titanium mesh plate was bent with the angle being equal to that formed between the orbital floor and the medial wall of the contralateral orbit. The shaped titanium mesh plate, with an allograft thickness of 0.55 mm, was implanted under the periosteum and overlaid the defect of the orbital floor fracture. The shaped titanium mesh plate was implanted under the periosteum and overlay the defect of the orbital floor fracture. And the implant was fixed to the inferior orbital rim with two titanium screws, which was in the symmetrical position to that of contralateral orbital floor. A forced duction test was performed immediately, and passive free eye movements were confirmed prior to the closure of the surgical incision. The incision was closed followed by a fixation of pressure bandage in the operated orbit for 48 hours. 

**Figure 1:**
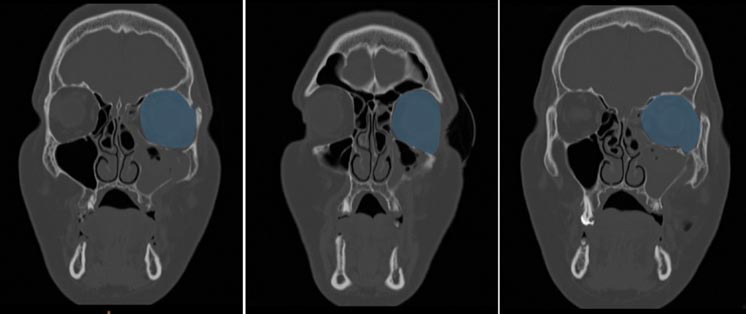
Preoperative computed tomography (CT) views of the traumatized left orbit in the coronal plane

**Figure 2:**
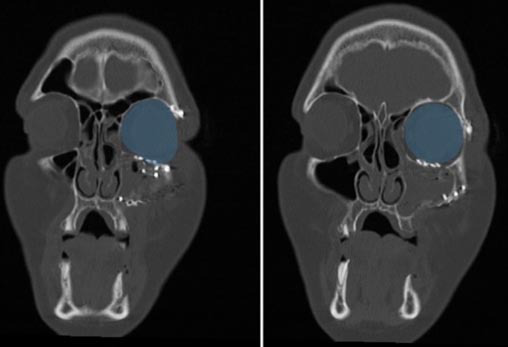
Postoperative computed tomography (CT) views of the reconstructed left orbit in the coronal plane

A 3D CT scan was performed in the first week after surgery to evaluate the quality of the reconstruction and the condition of the surrounding tissues to avoid any complications. He was re-evaluated 1 month later after binocular surgery. After the surgery, 3D CT scan photos were taken from the patient and measurements were made again, and the doctor checked the success of the treatment. All the 3D CT scan images were acquired by the CT scan machine (Emotion Somatom, Siemens, German) and processed by Materialise Mimics (Materialise NV©, Belgian). The volume of the orbits before and after treatment was determined by cross-sectional cutting techniques (Figures 1-2). 

Pre- and post-operative CT Digital Imaging and Communications in Medicine (DICOM) datasets were imported into Materialise Mimics v24.0 (Materialise NV, Leuven, Belgium) on Windows X64. Bony masks were generated via global thresholding, region growing, and manual mask editing (Edit Mask tool), with supplementary use of Split Mask and Smart Fill for complex regions. The final mask was converted to a 3D surface mesh using the Marching Cubes algorithm. Custom Python scripts within the Mimics Scripting module automated threshold and region-growing steps to enhance consistency. Orbital volumes were calculated by summing mask voxel volumes, and volume changes were determined by comparing pre- and post-operative models [ 15 ].

The results were analyzed using the Chi-square test and Student t-test in SPSS version 24 (SPSS Inc., IL, USA) *p*=< 0.05 was considered statistically significant.

## Results

Of all participants, 7% were females and 93% were ma-les. In patients with orbital trauma, orbital floor fracture had the highest frequency (37.7%) followed by the maxillary fracture (37.7%). Orbital wall fracture had the lowest frequency (32.6%). The frequency of maxillary, orbital floor, and orbital wall fracture was not significantly different in male patients (*p*> 0.05). However, the frequency of different orbital fracture types was different in females, and the frequency of maxillary and orbital floor fractures in females was greater than those in males, each accounting for 40% of fractures in females. Also, 20% of females had orbital wall fractures. However, the difference in the frequency of different orbital fractures was not significantly different between males and females (*p*= 0.82).

The frequency of orbital fracture was 50% on the right and 50% on the left side (*p*= 0.241). All females had left orbital fractures, and a right orbital fracture was not seen in any female patient. Also, 46.4% of males had left orbital fractures and 53.6% had right orbital fractures. The difference in this regard was not significant (*p*= 0.143). 

The independent t-test showed a significant difference in the volume of the traumatized orbit with the volume of the sound contralateral orbit and the traumatized orbit after corrective surgery (Table 1, *p*= 0.005). As shown, the volume of the traumatized orbit had an average of 2.48 cm^3^ difference from the sound orbit, and 1.42cm^3^ difference from the surgically corrected orbit. The mean volume of the sound and surgically corrected orbit was 27.27 and 27.67cm^3^, respectively; the independent t-test showed that the treatment was successful in all cases, and the volume of the treated orbit had no significant difference with the volume of the sound orbit (*p*= 0.38). 

**Table 1:** Results of independent t-test for comparison of the volume of the traumatized orbit with the volume of the sound contralateral orbit and the volume of the traumatized orbit after corrective surgery

Comparison	Mean difference	SD	SE	t	df	*p* Value
Traumatized and corrected orbit	-1.05967	1.91348	0.34935	-3.033	29	0.005
Corrected orbit and sound orbit	-0.39433	2.42298	0.44237	0.891	29	0.38

SD: Standard deviation; SE: Standard Error; t: t statistic; df: degrees of freedom; *p* Value (*p*)

## Discussion

Orbits are symmetrical bone structures whose specific three-dimensional shape and size are critical in maintaining the shape and position of the intraorbital soft tissues, as well as maintaining the normal protrusion of the eyeball [ 16 ]. Orbital reconstruction is always a challenge for surgeons due to anatomical complexity, small size, and exceptional importance of the eye for human life [ 17 ]. Despite the prevalence of post-traumatic orbital injuries, there is still a lack of consensus in the literature as to the optimal management pathway for this cohort of patients. This divergence encompasses not only the timing of surgery (i.e. how soon post-injury elective orbital reconstruction should take place) but also the operative approach and the implant type employed in the reconstruction of the orbital architecture [ 18 - 19 ]. 

This study assessed the changes in the orbital volume following reconstruction with alloplastic materials in patients with orbital trauma. The results showed no significant differences in the volume of the traumatized orbit with sound contralateral orbit and traumatized orbit after corrective surgery. Corrective surgery was successful in all patients and the orbital volume after treatment showed no significant difference from the volume of the sound contralateral orbit.

In our study, we used titanium mesh because it showed better results than bone grafts for orbital reconstruction according to Ellis and Tan [ 20 ], as it is malleable and adaptable to the shape of the defect, the most biocompatible of all available material, connective tissue can grow around and through the implant and prevent its migration. Also, Goldberg *et al*. [ 21 ] and Kelly *et al*. [ 22 ] showed that bone grafts were associated with donor site morbidity, variable degree of resorption, prolonged total operating time, postoperative pain, and scarring; they are usually rigid and cannot be bent to match the concave–convex shape of the orbital floor and provide less drainage from the orbit than with titanium.

Consistent with the results of our study, Chepurnyi *et al*. [ 23 ] demonstrated results with 0.74±0.6cm^3^ of mean difference between damaged and intact orbit after surgery. Beder *et al*. [ 4 ] showed that the mean difference between the intact/damaged orbital volumes was within the range of 1.35±0.86cm^3^ for pre-bent titanium mesh. Zhang *et al*. [ 24 ], Zimmerer *et al*. [ 25 ] and Chepurnyi *et al*. [ 23 ] demonstrated the mean difference between the intact/damaged orbital volumes for pre-bent titanium mesh was within the range of 1.6±2.4 and 1.9±1.4cm^3^.

The fracture of the lower third of the face is more common than that of the superior parts. However, the fracture of the superior parts is often accompanied by more serious complications. Orbital fracture often occurs due to motor vehicle accidents following direct trauma to the orbit. In such cases, fracture causes inward compression of the eyeball [ 26 ]. In orbital fracture, the broken segment(s) may be displaced externally (blow out) or internally (blow in). The blowout fracture of the orbital floor is the most common type of orbital fracture, which occurs through two mechanisms. The first mechanism is known as the hydraulic process, in which, the impact of any hard object or fluids with a diameter larger than that of the orbit increases the orbital pressure and leads to the fracture of the thinnest part of the wall, which is often the posterior-internal part of the inferior wall. In the second mechanism, the impact force is directly transferred through the orbital borders, causing an isolated fracture of the orbital floor. Orbital fracture is often associated with impaired vision, diplopia, ptosis, ectropion, canthus deformity, cranial involvement, anterior cranial damage, paresthesia, and esthetic impairments [ 26 ]. Correct diagnosis and prompt appropriate treatment are highly important to minimize such complications. 

The present results showed a higher frequency of orbital fracture in males than females (14:1) probably due to their active presence in the community and greater involvement in sports activities [ 27 ]; this is consistent with Ji *et al*.’s study [ 28 ] which was due to more physical activity of males. This ratio is variable in the literature ranging from 2:1 to 14:1 [ 5 , 29 ]. Such a high difference in involvement of males and females in the present study is attributed to the careless driving of males, not wearing a helmet, and unsafe use of motor vehicles, especially motorcycles and bikes [ 30 ]. In some other countries like the United States, street fights are the main cause of orbital fractures [ 31 - 32 ]. 

Although our calculated sample size of 73 patients met the requirements for statistical power, the number of female participants was low. This gender imbalance reflects the lower incidence of high-risk activities (e.g. motor vehicle accidents and fights) among women in Iran- as well as legal restrictions on motorcycle riding for women- and is therefore a true feature of our clinical population rather than recruitment bias.

Future multi-center studies with a larger sample size are required for a more comprehensive assessment of the causes and management techniques of orbital fractures in Iran.

## Conclusion

Corrective surgery with alloplastic materials can successfully regain the orbital volume in cases with orbital floor fracture with no significant difference with the volume of the sound contralateral orbit.
